# Mode of Applying Iodine to the Interior of Cysts

**Published:** 1876-05

**Authors:** 


A More Effectual Mode of Applying Iodine to the Interior of Certain Cysts.—Mr. Furneux Jordan, in some notes in the *Lancet*, remarks that he has found in practice two classes of scrotal hydroceles, in which the ordinary methods of treatment are either difficult to use or uncertain in their result. In boys and men there are occasionally encysted hydroceles of the testis, or the cord, which continue to increase in size, or in which treatment is urgently requested. In such cases, except in early infancy, acupuncture, or the use of a fine trocar, often fails to cure. The walls of the cysts are usually thin, and collapse so much when their contents are withdrawn that the injection of a fluid is uncertain. The end of the canula may be outside the cyst, and the iodine solution be consequently injected into the connective tissue at its exterior. In such cases the following is a reliable method of treatment: "The cyst being well isolated, made tense, and brought near the surface, I pass through its centre a stout needle, armed with silk, and leave the threads hanging. The fluid quickly oozes away, especially if a little traction be made on the threads. I then, at one opening, wet the threads with iodine liniment (liniment because the quantity required is so limited) and draw the threads so as to leave moistened portions within the cyst. A little gentle friction will help to spread the iodine thoroughly over the lining membrane of the cavity. An hour later freshly moistened portions may again be drawn through, if the cyst be large, or if other methods of treatment have failed. On the other hand, in a very small cyst, a single thread, moistened and kept in one hour, will suffice.

"Another class of cases are those of simple vaginal hydrocele, in which the injection of iodine and other ordinary methods of treatment are unsuccessful. An interesting case will best convey what I wish to say. A young man had a moderate-sized hydrocele. Trocar puncture and acupuncture repeated a few times failed, and consequently iodine tincture (undiluted) was injected. In a few weeks the collection had reached its old size. A silver-wire seton was then put in; while in the cyst remained empty, but its removal was followed by reappearance of the fluid. I then, at three o'clock, passed through the cavity a double silk thread at two spots. In a few minutes, when all the fluid had oozed out, I drew the threads, moistened with iodine liniment, into the serous cavity. I directed him to repeat the process in an hour. He was so anxious to get well—he was shortly to be married—that he moistened the threads four times in six hours. At midnight the effects had become so sharp that he was glad to remove the threads, as he had been directed. He remained at home one day only, and was shortly and permanently well.

"I venture to believe that no kind of hydrocele will resist this method of applying iodine, and consequently that the setting up of suppuration, even as a last resort, can rarely be necessary."

